# Nonandrogenic Anabolic Hormones Predict Risk of Frailty: European Male Ageing Study Prospective Data

**DOI:** 10.1210/jc.2017-00090

**Published:** 2017-05-09

**Authors:** Agnieszka Swiecicka, Mark Lunt, Tomás Ahern, Terence W. O’Neill, György Bartfai, Felipe F. Casanueva, Gianni Forti, Aleksander Giwercman, Thang S. Han, Michael E. J. Lean, Neil Pendleton, Margus Punab, Jolanta Slowikowska-Hilczer, Dirk Vanderschueren, Ilpo T. Huhtaniemi, Frederick C. W. Wu, Martin K. Rutter, for the EMAS Study Group

**Affiliations:** 1 Andrology Research Unit, Division of Diabetes, Endocrinology and Gastroenterology, School of Medical Sciences, Faculty of Biology, Medicine and Health, University of Manchester, Manchester Academic Health Science Centre, Manchester M13 9WL, United Kingdom; 2 Arthritis Research UK Centre for Epidemiology, Faculty of Biology, Medicine and Health, University of Manchester, Manchester Academic Health Science Centre, Manchester M13 9PT, United Kingdom; 3 National Institute for Health Research, Musculoskeletal Biomedical Research Unit, Central Manchester National Health Service Foundation Trust, Manchester M13 9WL, United Kingdom; 4 Department of Obstetrics, Gynecology and Andrology, Albert Szent-György Medical University, H6725 Szeged, Hungary; 5 Department of Medicine, Santiago de Compostela University, Complejo Hospitalario Universitario de Santiago, CIBER de Fisiopatología Obesidad y Nutricion, Instituto Salud Carlos III, 15076 Santiago de Compostela, Spain; 6 Sexual Medicine and Andrology Unit, Department of Experimental and Clinical Biomedical Sciences “Mario Serio,” University of Florence, 50139 Florence, Italy; 7 Reproductive Medicine Center, Malmö University Hospital, University of Lund, SE-205 02 Malmö, Sweden; 8 Institute of Cardiovascular Research, Royal Holloway University of London and Ashford and St. Peter’s National Health Service Foundation Trust, Surrey KT16 0PZ, United Kingdom; 9 Department of Human Nutrition, University of Glasgow, Glasgow G31 2ER, United Kingdom; 10 Division of Neuroscience and Experimental Psychology, School of Biological Sciences, University of Manchester, Hope Hospital, Salford M6 8HD, United Kingdom; 11 Andrology Unit, United Laboratories of Tartu University Clinics, 50406 Tartu, Estonia; 12 Department of Andrology and Reproductive Endocrinology, Medical University of Lodz, 90-419 Lodz, Poland; 13 Department of Andrology and Endocrinology, Katholieke Universiteit Leuven, BE-3000 Leuven, Belgium; 14 Department of Surgery and Cancer, Institute of Reproductive and Developmental Biology, Imperial College London, Hammersmith Campus, London W12 0NN, United Kingdom; 15 Department of Physiology, Institute of Biomedicine, University of Turku, FI-20520 Turku, Finland; 16 Manchester Diabetes Centre, Central Manchester University Hospitals NHS Foundation Trust, Manchester Academic Health Science Centre, Manchester M13 9WL, United Kingdom

## Abstract

**Context:**

Low levels of nonandrogenic anabolic hormones have been linked with frailty, but evidence is conflicting and prospective data are largely lacking.

**Objective:**

To determine associations between nonandrogenic anabolic hormones and prospective changes in frailty status.

**Design/Setting:**

A 4.3-year prospective observational study of community-dwelling men participating in the European Male Ageing Study.

**Participants:**

Men (n = 3369) aged 40 to 79 years from eight European centers.

**Main Outcome Measures:**

Frailty status was determined using frailty phenotype (FP; n = 2114) and frailty index (FI; n = 2444).

**Analysis:**

Regression models assessed relationships between baseline levels of insulinlike growth factor 1 (IGF-1), its binding protein 3 (IGFBP-3), dehydroepiandrosterone sulfate (DHEA-S), 25-hydroxyvitamin D (25OHD), and parathyroid hormone (PTH), with changes in frailty status (worsening or improving frailty).

**Results:**

The risk of worsening FP and FI decreased with 1 standard deviation higher IGF-1, IGFBP-3, and 25OHD in models adjusted for age, body mass index, center, and baseline frailty [IGF-1: odds ratio (OR) for worsening FP, 0.82 (0.73, 0.93), percentage change in FI, −3.7% (−6.0, −1.5); IGFBP-3: 0.84 (0.75, 0.95), −4.2% (−6.4, −2.0); 25OHD: 0.84 (0.75, 0.95); −4.4%, (−6.7, −2.0)]. Relationships between IGF-1 and FI were attenuated after adjusting for IGFBP-3. Higher DHEA-S was associated with a lower risk of worsening FP only in men >70 years old [OR, 0.57 (0.35, 0.92)]. PTH was unrelated to change in frailty status.

**Conclusions:**

These longitudinal data confirm the associations between nonandrogenic anabolic hormones and the changes in frailty status. Interventional studies are needed to establish causality and determine therapeutic implications.

## Teaser

We studied the endocrine risk factors for frailty and found significant longitudinal relationships between higher IGF-1, IGFBP-3, and vitamin D and lower risk of worsening frailty in aging men.

Frailty describes a state of increased vulnerability to stressors and reduced homeostatic reserve in the elderly that is associated with adverse outcomes such as loss of mobility, functional disabilities, dependency, and death (1, 2). With the rapidly aging population, these frailty-related problems present an increasing challenge to health care systems worldwide. Better understanding of the underlying causes and natural history of frailty can enable early identification of at-risk individuals, development of new treatments, and instigation of prevention strategies.

The pathophysiology of frailty has been linked with dysfunctions in multiple physiological systems but the mechanisms remain poorly understood. Decline in muscle mass and function is thought to be central to the development of frailty, and because anabolic hormones are one of the key factors responsible for muscle growth and repair, endocrine dysregulation has been suggested as a potential etiological factor for frailty. This is further supported by clinical sequelae of various endocrinopathies that share common features with those of frailty in both men and women.

Several studies have investigated associations of anabolic hormones with muscle function and physical performance but relatively few have focused on frailty (3–7). These studies have been mostly cross-sectional and prospective data are limited. For example, studies of insulinlike growth factor 1 (IGF-1) have been negative, but results may have been influenced by low statistical power and overadjustment bias (3, 4). Prior results on 25-hydroxyvitamin D (25OHD) have also been conflicting (3, 5, 8, 9), and studies focusing on dehydroepiandrosterone sulfate (DHEA-S) have been underpowered (10, 11). The results of studies examining the associations between testosterone and frailty are largely inconsistent (12, 13); we have explored the relationship between testosterone and frailty in our population and the findings are presented separately.

In this study, we hypothesized that nonandrogenic anabolic hormone levels would be related to changes in frailty status. Using the population-based European Male Ageing Study (EMAS), we sought to determine prospective associations between a number of nonandrogenic anabolic hormones and changes in frailty status.

## Methods

### Subjects

The subjects were participants in the EMAS, the details of which have been described previously (14). Briefly, 3369 men aged 40 to 79 were recruited between 2003 and 2005 from population-based sampling frames in eight European centers. The participants completed a series of questionnaires, anthropometric measurements, and physical and cognitive functional assessments, and a fasting blood sample was collected. Ethical approval was obtained in accordance with the local requirements.

Participants were recontacted after a minimum of 4 years (median, 4.3 years); the methods of data collection at follow-up were the same as in the baseline study. During the follow-up period, 193 (6%) men died and 440 (13%) were lost to follow-up.

The longitudinal analysis was restricted to men with complete frailty data at baseline and follow-up. We excluded participants with self-reported adrenal or pituitary disease or the use of medications affecting the somatotrophic axis (dopamine agonist, recombinant human growth hormone, somatostatin analog), parathyroid hormone (PTH)/25OHD levels (vitamin D and/or calcium supplements/analogs), or DHEA-S levels (DHEA-S supplements).

### Assessment of hormone predictors

A fasting morning (before 10:00 am) venous blood sample was used for all hormone measurements as previously described (6, 15) (see Supplemental Data for details).

### Clinical assessments

Participants were asked about lifestyle, general health, and comorbidities. The interviewer-assisted questionnaire included the Medical Outcomes Study 36-item short form survey (16), the Physical Activity Scale for the Elderly (17), Beck Depression Inventory (18), and International Prostate Symptoms Score (19). Physical function was assessed by the Reuben Physical Performance Test (20) and the Tinetti balance and postural stability index (21). The Rey–Osterrieth Complex Figure test (22), the Camden Topographical Recognition Memory test (23), and the Digit-Symbol Substitution test (24) were used to assess cognitive function. We assessed height, weight, waist circumference, middle upper arm and calf circumferences, and skin-fold thickness at several body sites.

### Assessment of frailty

Frailty was characterized by the two widely used approaches: frailty phenotype (FP) and frailty index (FI).

The EMAS FP was first developed in 2011 as an adaptation from the Cardiovascular Health Study (2) and was based on five criteria: sarcopenia, exhaustion, slowness, weakness, and low activity (see Supplemental Table 1 for details). Individuals with three or more criteria were classed as frail, those with one or two criteria as prefrail, and those with none as robust. The EMAS FP has been internally validated by showing that it predicts adverse health outcomes, including falls and death (25).

The EMAS FI comprises 39 health deficits (symptoms and signs, functional and cognitive impairments; Supplemental Table 2) known to accumulate with age and associated with adverse health outcomes. The deficit variables were derived from the Medical Outcomes Study 36-item short form survey and Beck Depression Inventory questionnaires, physical performance and cognitive tests, and self-reported comorbidities (20–23). The EMAS FI was created using a standardized procedure (26) and calculated as a ratio of deficits present to the total number of possible deficits. Binary variables were coded as 0 or 1 (absent/present), and intermediate responses (*e.g.*, sometimes/maybe) were coded as 0.5. Continuous variables were dichotomized based on the distribution of participants’ scores; cut-points were set at the worst performing 10th centile. Individuals with >20% missing data were excluded.

### Statistical analysis

Descriptive statistics are presented as the mean ± standard deviation (SD) or n (%), and statistical significance of between-group differences was assessed using analysis of variance.

#### Frailty phenotype models

Change in frailty was defined using transitions in frailty states between baseline and follow-up. The transitions considered were: worsening frailty (robust or prefrail at baseline progressing to prefrail or frail at follow-up; referent category, persistent robust and persistent prefrail) and improving frailty (prefrail or frail at baseline transitioning to robust or prefrail state at follow-up; referent category, persistent frail and prefrail).

Logistic regression models determined relationships between an individual predictor (hormone at baseline) and outcome (transition in frailty state). Each potential endocrine predictor was considered as an untransformed value standardized as a *z* score [(raw score – mean)/SD] to allow comparison of results between endocrine predictors with different units of measurement. We adjusted all models for baseline frailty to account for the heterogeneity of baseline frailty status. Models were then further adjusted for variables significantly correlated with the hormonal predictors, such as age and body mass index (BMI), and center. In further analyses, the effects of additional adjustments for other potential confounders, such as smoking, alcohol use, education, physical activity, diabetes and cardiovascular disease (CVD), and other hormones were also explored. Additionally, all analyses in which PTH and 25OHD were the main predictor variables were adjusted for 25OHD and PTH levels, respectively. The results were displayed as odds ratios (OR) with 95% confidence intervals (CIs) for change in frailty status associated with a 1 SD difference in baseline hormone level.

#### FI models

In view of the right skewing of the FI variable, relationships between baseline hormone level and FI at follow-up were assessed using a negative binomial regression. The FI variable was converted to a 0- to 39-count scale where “0” represented no deficits and “39” represented the maximum number of deficits. Analyses were serially adjusted for baseline FI (model 1), age (model 2), center (model 3), and BMI (model 4). As earlier, endocrine predictors were standardized as *z* scores. The results were presented as percentage change (95% CI) in FI associated with a 1 SD difference in baseline hormone level (negative values indicating improving frailty and positive values indicating worsening frailty during follow-up).

To assess for potential effect of age on the relationships between hormones and frailty, we performed an exploratory analysis introducing an interaction term (baseline hormone × age category) to the fully adjusted model. The “age category” represented four age bands: 40 to 49, 50 to 59, 60 to 69, and 70 to 79 years.

All analyses were performed using Stata 13 SE software (StataCorp, College Station, TX).

## Results

Of the 3369 men who participated in EMAS, 2444 men remained in the FI analysis and 2114 in the FP analysis after excluding those with known pituitary or adrenal disease (n = 29), relevant medication use (n = 47), missing FI (n = 556) or FP (n = 226) data, and failure to show up for follow-up assessment (n = 623) (Supplemental Fig. 1). Compared with the main analytical sample, men lost to follow-up (n = 435) were older and had a higher prevalence of smoking, depression, diabetes, and frailty at baseline (Supplemental Tables 3 and 4). This was the case also for the men who died (n = 188), with addition of a higher systolic blood pressure, creatinine, and waist-to-hip ratio in this group when compared with the analytical sample.

### Worsening frailty

Of 1589 men who were robust at baseline, 390 became prefrail and 20 became frail at follow-up. Among 505 men who were prefrail at baseline, 49 progressed to frailty. Therefore, in total, 459 men presented with worsening frailty at follow-up (Supplemental Fig. 2).

### Improving frailty

One hundred ninety-two men who were prefrail and two who were frail at baseline became robust at follow-up. Additionally, 12 men who were frail at baseline transitioned to the prefrail state at follow-up. Therefore, in total, an improvement in frailty status was observed in 206 men (Supplemental Fig. 2).

#### Population characteristics

Baseline characteristics are shown in Table 1. The men had a mean age of 59 years and a mean BMI of 28 kg/m^2^. Six percent were known to suffer from diabetes, 21% from depression, and 33% reported a history of CVD. Twenty percent admitted current smoking.

**Table 1.** **Baseline Characteristics of the Study Population**

Baseline Parameter	Mean ± SD or n (%)
N	2444
Age, y	59 ± 11
BMI, kg/m^2^	28 ± 4
WHR	0.98 ± 0.06
Waist circumference, cm	98 ± 11
Smoking, n (%)	474 (20%)
Frequent alcohol use, n (%)	561 (23%)
Below degree education	1731 (71%)
Systolic BP, mm Hg	146 ± 20
Diastolic BP, mm Hg	87 ± 12
Creatinine, μmol/L	92 ± 28
PASE score	202 ± 89
Severe depression (BDI bands 4–6), n (%)	82 (4%)
Mild depression (BDI bands 2–3), n (%)	398 (17%)
CVD	702 (33%)
Diabetes, n (%)	153 (6%)
DHEA-S, µmol/L	4.6 ± 2.7
PTH, pg/mL	28.3 ± 14.2
25OHD, ng/mL	25.9 ± 12.9
IGF-1, µg/L	135 ± 42.8
IGFBP-3, µg/mL	4.5 ± 1.0

Abbreviations: BDI, Beck Depression Inventory score, BP, blood pressure; PASE, Physical Activity Scale for the Elderly; WHR, waist-to-hip ratio.

Compared with men who did not experience any change in frailty status, men whose frailty status deteriorated were older (61 vs 57 years), were less active physically, had lower BMI, and had a higher prevalence of diabetes and CVD (Table 2). Their baseline levels of DHEA-S, IGF-1, and its binding globulin 3 (IGFBP-3) were significantly lower than those whose frailty status remained stable.

**Table 2.** **Baseline Parameters Stratified by Frailty Transition Group, as Assessed by Frailty Phenotype Derived From the Cardiovascular Health Study**

Baseline Parameter	Worsening Frailty*^a^*	Persistent Robust and Persistent Prefrail	*P* Value	Improving Frailty*^b^*	Persistent Frail and Persistent Prefrail	*P* Value
N	459	1443		206	270	
Age, y	61 ± 11	57 ± 10	<0.001	59 ± 10	64 ± 10	<0.001
BMI, kg/m^2^	27.3 ± 4.0	27.7 ± 4.0	0.029	27.7 ± 4.2	27.2 ± 5.0	0.105
WHR	0.98 ± 0.06	0.98 ± 0.06	0.309	0.99 ± 0.06	0.98 ± 0.07	0.856
Waist circumference, cm	98.1 ± 10.8	97.9 ± 10.7	0.857	99 ± 11.6	98.4 ± 13.1	0.59
Smoking, n (%)	101 (22)	271 (19)	0.252	45 (22)	63 (23)	0.517
Frequent alcohol use, n (%)	116 (25)	333 (23)	0.341	50 (24)	56 (21)	0.515
Below degree education, n (%)	329 (72)	1014 (70)	0.564	157 (76)	204 (76)	0.868
Systolic BP, mm Hg	145 ± 20	145 ± 20	0.735	145 ± 22	148 ± 23	0.138
Diastolic BP, mm Hg	87 ± 11	87 ± 12	0.46	89 ± 13	86 ± 12	0.023
Creatinine, μmol/L	91 ± 17	92 ± 33	0.988	90 ± 17	92 ± 17	0.209
PASE score	188 ± 82	214 ± 87	<0.001	156 ± 93	139 ± 92	0.049
Severe depression (BDI bands 4–6), n (%)	8 (2)	35 (2)	0.112	10 (5)	21 (8)	0.247
Mild depression (BDI bands 2–3), n (%)	83 (18)	212 (15)	0.214	49 (24)	74 (37)	0.267
CVD, n (%)	178 (29)	281 (22)	0.001	79 (38)	127 (48)	0.033
Diabetes, n (%)	38 (8)	69 (5)	0.013	11 (5)	24 (9)	0.321
DHEA-S, µmol/L	4.5 ± 2.8	4.8 ± 2.7	0.005	4.4 ± 2.4	4.0 ± 2.7	0.008
PTH, pg/mL	28.7 ± 12.8	28.1 ± 14.9	0.208	30.0 ± 14.9	29.3 ± 11.8	0.779
25OHD, ng/mL	25.5 ± 13.8	26.2 ± 12.7	0.066	25.8 ± 13.4	24.1 ± 13.0	0.117
IGF-1, µg/L	128.8 ± 38.9	138.5 ± 44.2	<0.001	131.2 ± 42.8	128.4 ± 46.2	0.267
IGFBP-3, μg/mL	4.4 ± 1.0	4.6 ± 1.0	<0.001	4.4 ± 1.1	4.3 ± 1.0	0.068

Data are expressed as mean ± SD for continuous variables or as number (percentage) for binary categorical variables. *P* values were calculated using baseline parameters and using analysis of variance.

Abbreviations: BDI, Beck Depression Inventory score; BP, blood pressure; PASE, Physical Activity Scale for the Elderly; WHR, waist-to-hip ratio.

^a^ Robust or prefrail men at baseline progressing to prefrail or frail state at follow-up.

^b^ Prefrail or frail men at baseline transitioning to robust or prefrail state at follow-up.

Men whose frailty status improved were younger (59 vs 64 years) and had higher diastolic blood pressure and a lower prevalence of CVD when compared with men who remained prefrail or frail throughout the study period (Table 2). Baseline levels of anabolic hormones were numerically higher in men who experienced improvement in frailty, although the differences were not statistically significant.

#### Hormonal predictors of worsening frailty

Higher baseline levels of IGF-1 and IGFBP-3 were associated with a lower likelihood of worsening frailty status as assessed by FP and FI in baseline frailty–adjusted models with and without additional adjustment for age, BMI, and center (Tables 3 and 4, models 1 through 4). When the IGF-1 analysis was also adjusted for IGFBP-3, IGF-1 was no longer associated with lower risk of worsening FI (Supplemental Table 5). Similarly, IGFBP-3 was no longer an independent predictor of FI and FP after adjustment for IGF-1.

**Table 3.** **Association Between Baseline Level of Anabolic Hormone and 4-Year % Change in FI: Models and Adjustments**

Baseline Parameter	N												
		Model 1: Baseline Frailty			Model 2: Baseline Frailty and Age			Model 3: Baseline Frailty, Age, and Center			Model 4: Baseline Frailty, Age, Center, and BMI		
		% Change*^a^*	95% CI	*P* Value	% Change*^a^*	95% CI	*P* Value	% Change*^a^*	95% CI	*P* Value	% Change*^a^*	95% CI	*P* Value
IGF-1	2426	−7.2	−9.3, −5.0	<0.001	−4.1	−6.2, −1.7	0.001	−3.7	−6.0, −1.5	0.001	−3.7	−6.0, −1.5	0.001
IGFBP-3	2428	−8.2	−10.5, −5.8	<0.001	−4.8	−6.9, −2.6	<0.001	−3.5	−5.7, −1.4	0.002	−4.2	−6.4, −2.0	<0.001
DHEA-S	2428	−6.0	−8.2, −3.6	<0.001	1.0	−1.5, 3.7	0.437	−0.1	−2.7, 2.4	0.917	0.2	−2.3, 2.8	0.852
PTH*^b^*	2429	2.2	0.1, 4.5	0.059	1.3	−0.8, 3.6	0.230	1.3	−0.8, 3.6	0.231	0.9	−1.2, 3.1	0.402
25OHD*^c^*	2347	−3.3	−5.6, −1.0	0.006	−4.7	−6.9, −2.5	<0.001	−4.7	−7.0, −2.4	<0.001	−4.4	−6.7, −2.0	<0.001

^a^ Change (% change/4 years) in FI per SD increase in anabolic hormone level. Negative % change means that the baseline hormone level is associated with improvement of frailty status, whereas positive % change means that the hormone is associated with worsening of frailty status.

^b^ Models 3and 4 additionally adjusted for baseline 25OHD level.

^c^ Models 3 and 4 additionally adjusted for baseline PTH level.

**Table 4.** **Multivariable-Adjusted OR (95% CI) for Worsening Frailty Phenotype Associated With Baseline Hormonal Predictor: Models and Adjustments**

Baseline Parameter	N												
		Model 1: Baseline Frailty			Model 2: Baseline Frailty and Age			Model 3: Baseline Frailty, Age, and Center			Model 4: Baseline Frailty, Age, Center, and BMI		
		OR	95% CI	*P* Value	OR	95% CI	*P* Value	OR	95% CI	*P* Value	OR	95% CI	*P* Value
IGF-1	1885	0.77	0.68, 0.86	<0.001	0.85	0.75, 0.96	0.008	0.82	0.73, 0.93	0.002	0.82	0.73, 0.93	0.002
IGFBP-3	1888	0.81	0.73, 0.90	<0.001	0.90	0.80, 1.01	0.070	0.84	0.74, 0.94	0.003	0.84	0.75, 0.95	0.006
DHEA-S	1891	0.87	0.78, 0.97	0.012	1.07	0.95, 1.22	0.259	1.07	0.94, 1.21	0.299	1.06	0.93, 1.20	0.390
PTH*^a^*	1890	1.05	0.95, 1.16	0.329	1.03	0.93, 1.14	0.588	1.00	0.90, 1.12	0.938	1.01	0.90, 1.13	0.860
25OHD*^b^*	1828	0.92	0.83, 1.03	0.162	0.89	0.80, 1.00	0.053	0.86	0.76, 0.97	0.013	0.84	0.75, 0.95	0.007

^a^ Models 3 and 4 additionally adjusted for baseline 25OHD level.

^b^ Models 3 and 4 additionally adjusted for baseline PTH level.

Higher DHEA-S levels were associated with lower risk of worsening frailty status as assessed by both FP and FI (Tables 3 and 4). However, statistical significance was lost after adjusting for age in both analyses.

Baseline PTH was unrelated to change in frailty status in any model (Tables 3 and 4).

Higher 25OHD levels were associated with lower risk of worsening frailty status as assessed by FI and FP (Tables 3 and 4). Although the relationship between 25OHD level and FP was not significant in the models adjusted for baseline frailty and age, the point estimate for the OR was similar to that in the fully adjusted model (0.92 vs 0.84, Table 4). Further adjustment of the models for other putative confounders that were not components of FI or FP but correlated with endocrine predictors—such as smoking, alcohol use, education, physical activity, diabetes, CVD, testosterone, and estradiol—did not alter the results.

#### Hormonal predictors of improving frailty phenotype

Higher baseline levels of IGFBP-3 were associated with a greater probability of improving frailty phenotype in only the model adjusted for baseline frailty status (Table 5). This relationship became nonsignificant after adjusting for age. Higher vitamin D levels were associated with greater probability of improved frailty in baseline frailty–, age-, center-, and BMI-adjusted models (Table 5). This relationship, however, was rendered nonsignificant following adjustment for the presence of diabetes (Supplemental Table 7).

**Table 5.** **Multivariable-Adjusted OR (95% CI) for Improving Frailty Phenotype Associated With Baseline Hormonal Predictor: Models and Adjustments**

Baseline Parameter	N												
		Model 1: Baseline Frailty			Model 2: Baseline Frailty and Age			Model 3: Baseline Frailty, Age, and Center			Model 4: Baseline Frailty, Age, Center, and BMI		
		OR	95% CI	*P* Value	OR	95% CI	*P* Value	OR	95% CI	*P* Value	OR	95% CI	*P* Value
IGF-1	471	1.08	0.91, 1.29	0.377	0.96	0.79, 1.15	0.643	0.94	0.78, 1.15	0.555	0.94	0.77, 1.14	0.539
IGFBP-3	471	1.21	1.00, 1.46	0.049	1.06	0.87, 1.29	0.579	1.04	0.85, 1.28	0.686	1.02	0.83, 1.25	0.863
DHEA-S	471	1.19	0.98, 1.45	0.072	0.91	0.73, 1.13	0.396	0.94	0.75, 1.17	0.589	0.95	0.76, 1.18	0.640
PTH*^a^*	472	1.03	0.84, 1.26	0.775	1.08	0.87, 1.33	0.476	1.09	0.87, 1.37	0.452	1.08	0.86, 1.36	0.481
25OHD*^b^*	459	1.14	0.94, 1.37	0.172	1.18	0.97, 1.44	0.099	1.25	0.99, 1.56	0.051	1.27	1.01, 1.58	0.039

^a^ Models 3 and 4 additionally adjusted for baseline 25OHD level.

^b^ Models 3 and 4 additionally adjusted for baseline PTH level.

#### Interaction with age

In a secondary analysis, there was evidence of age-related differences in the relationships between DHEA-S and worsening FP. In men aged >70 years, higher baseline DHEA-S levels were associated with a lower risk of worsening FP [OR, 0.57 (0.35, 0.92), *P* = 0.021], but this relationship was not observed in younger men (*P* value for interaction of 0.001). The association in older men remained significant after further adjustment for depression score and diabetes. There were no significant age-related interactions in the relationships between other hormones and change in FP or FI (not shown).

## Discussion

Our study has shown robust longitudinal multivariable-adjusted relationships between higher baseline levels of IGF-1, IGFBP-3, and vitamin D and lower 4-year risks of worsening frailty status in middle-aged and older community-dwelling European men. In models adjusted for baseline frailty status, higher levels of DHEA-S were associated with lower risk of worsening frailty status but statistical significance was lost on age adjustment. We also showed that baseline PTH was unrelated to changes in frailty status in any model. Importantly, the associations between hormone levels and frailty showed consistency regardless of whether frailty was assessed by FP or FI. These data highlight potential mechanisms of frailty and identify possible modifiable targets for intervention.

### Comparison with prior studies, including mechanistic explanations

#### IGF-1 and IGFBP-3

Our findings contrast with the results from the longitudinal study of Yeap *et al.* (4) of 1484 men >70 years of age that showed no significant multivariable-adjusted relationship between IGF-1 and IGFBP-3 and incident frailty as assessed by questionnaire. Consistent with our data, Yeap *et al.* did show significant univariate associations between higher IGF-1 and IGFBP-3 levels and lower risks for incident frailty. However, statistical significance was lost after adjusting for age, BMI, smoking, diabetes, and fasting status. This loss of significance could perhaps be explained by covariates such as BMI being on the causal pathway linking hormone levels to frailty. However, when we also adjusted for baseline levels of BMI, diabetes, and depression, higher levels IGF-1 and IGFBP-3 were still significantly related to a lower risk of frailty progression (Supplemental Tables 5 and 6). Therefore, overadjustment bias cannot fully explain the discrepant results. It seems likely that sample size, the restricted and older age range (>70 years), and the quartile modeling strategy adopted by Yeap *et al.* (4) may have limited the statistical power to show significant relationships. Our study adds important data by assessing frailty through clinical objective assessments (not simply questionnaires) based on validated methods, in a larger cohort, over a wider age range and in a younger group capturing earlier frailty transitions and having a lower risk of “healthy survivor” bias.

Similarly, our results contrast with those from a study of 1271 men and women >65 years of age who were participants in the Longitudinal Aging Study Amsterdam (3). In that study, the significance of the univariate relationship between lower IGF-1 levels and a higher risk for incident frailty was lost after adjusting for age, sex, medication, obesity, physical activity, and chronic disease. The lack of significance in fully adjusted models may have been explained by limited statistical power and overadjustment but may also have been influenced by the use of a nonvalidated measure of frailty and the absence of a sex-stratified analysis.

Our results are biologically plausible in light of previous research, highlighting the central role of IGF-1 in mediating muscle growth and repair across the lifespan (27). Despite well-documented effects of IGF-1 on muscle physiology in animal models, human studies have demonstrated conflicting results concerning associations of IGF-1 with muscle mass and physical performance (28, 29). One possible reason for these conflicting results might be the largely overlooked role of IGF binding proteins in mediating the bioavailability of IGF-1. We have studied the biologically most important IGF binding protein in humans, IGFBP-3. This is bound to IGF-1 in a complex that not only serves to extend the half-life of IGF-1 but also acts as an IGF-1 reservoir. IGFBP-3 is also thought to exert independent growth factor properties on cells as well as potentiate the effects of IGF-1 (30). In our analysis, the relationship between IGF-1 and frailty was largely attenuated by IGFBP-3 adjustment, suggesting a likely mediating rather than confounding role of IGFBP-3 in the IGF-1/frailty association.

#### Vitamin D

Our group (6) and others (3, 31) have described significant cross-sectional relationships between low vitamin D and frailty in the general population. For example, the InCHIANTI study showed that the risk of prevalent frailty was fourfold higher in men with low vitamin D levels compared with men with normal levels (5). In the present study, the largest prospective study reported to date, we report strong multivariable-adjusted relationships between higher levels of vitamin D level and reduced risk of frailty progression.

Several smaller studies have assessed the prospective relationship between vitamin D and incident frailty/reduced physical performance and have generated differing results, some positive (8, 32) and some negative (9, 33). Comparison between studies is limited particularly because of the different frailty definitions adopted and marked variation in modeling strategy, including covariate adjustment.

The largest of these prior studies involved 1267 community-dwelling men in which baseline vitamin D level did not predict incident frailty defined by the Fried FP criteria (9). The analysis was adjusted for a large number of confounders and the predictor (vitamin D level) was not considered as a continuous variable, which might have adversely affected the statistical power.

There are several potential mechanisms through which low vitamin D levels could contribute to frailty. Low vitamin D levels have been linked to altered muscle protein synthesis, decreased muscle strength, sarcopenia, worsening physical performance, and falls (34). These effects of low vitamin D on muscle are thought to be partially mediated by raised proinflammatory cytokines such as interleukin (IL)-12 and IL-2 (5, 34). Low vitamin D could also contribute to frailty indirectly through secondary hyperparathyroidism. In our analysis, however, adjustment for PTH level did not appear to alter the nature of the relationship between 25OHD and frailty.

Our prospective data are also supported by results of clinical trials and meta-analyses that indicate beneficial effects of vitamin D supplementation on muscle function, especially in older individuals with vitamin D deficiency (35). However, no trials to date have reported the effects of vitamin D therapy on the development of frailty.

#### PTH

This hormone has potential mechanistic links to frailty because it has been implicated in the development of neuromuscular dysfunction and has effects on muscle protein turnover and energy metabolism (36). The direct relationship of PTH with frailty has been explored in a small number of cross-sectional studies but, to the best of our knowledge, there have been no prior prospective studies. One prospective study reported an association of higher PTH levels with accelerated muscle loss, which can contribute to frailty (37).

We previously reported a significant cross-sectional association of baseline PTH level and prevalent frailty, which persisted following multiple adjustments including vitamin D (6). Shardell *et al.* (5) reported that elevated PTH was strongly associated with all individual components of the FP in elderly men, except low activity levels.

We now showed that baseline PTH was unrelated to change in frailty status in multiple adjusted models. However, in view of the strong potential mechanistic arguments, additional prospective studies may be valuable to further assess the relationship between PTH and frailty.

#### DHEA-S

We found that higher baseline DHEA-S levels were associated with a lower risk of worsening frailty. This association became nonsignificant after adjusting for age, BMI, and center, except for men older than 70 years, in whom higher DHEA-S level remained predictive of lower risk of frailty progression in a fully adjusted model.

Only two small studies (n = 254 and n = 416) have previously reported relationships between DHEA-S levels and incident frailty (10, 11). Both studies enrolled elderly community-based men and showed similar relationships to our study. Our data confirm these previous findings in a much larger cohort of middle-aged and elderly men.

Several potential pathways could link low DHEA-S with frailty. DHEA-S may have an anabolic effect on skeletal muscles, which has been suggested to be age-dependent and vary across lifespan (38) and, more recently, immunomodulatory and neuroprotective effects of this hormone have been described (39). However, results of DHEA-S supplementation in nonfrail adults have been disappointing and there is insufficient evidence to support the use of DHEA-S in the frail.

### Strengths and limitations

Our study has several important strengths, which include (1) use of a well-defined, longitudinal, community-based multicenter cohort, (2) a large sample size with adequate power to provide conclusive results, (3) use of standardized methods to assess hormone levels, and (4) use of two well-validated frailty models providing internally consistent results. We acknowledge some limitations, including the response rate for participation at baseline, which was 41%. Although this is comparable to other large epidemiological studies, the prevalence of frailty at baseline might have been overestimated or underestimated through selection. Additionally, 435 men were lost to follow-up and therefore the true incidence of frailty has probably been underestimated. Because this would tend to bias the results toward the null, the reported strength of our associations is likely to be conservative. Finally, our analysis is based on the results of single hormone measurements that do not capture pulsatile hormone variation and could attenuate regression coefficients to the null through regression dilution bias.

### Conclusion

We showed significant longitudinal age-adjusted relationships between higher levels of IGF-1, IGFBP-3, and vitamin D and lower risks of worsening frailty in men. In unadjusted analyses, we also showed that higher levels of DHEA-S were associated with lower risk of worsening frailty. These robust findings from a large well-characterized community-based cohort enhance our understanding of the etiology of frailty and suggest potential for therapeutic interventions that could shape new treatment strategies and public health policies aimed at increasing health span, independence, and well-being in older age.
