# Supplementary material for: Nonandrogenic Anabolic Hormones Predict Risk of Frailty: European Male Ageing Study Prospective Data
Source: J Clin Endocrinol Metab. 2017 May 9;102(8):2798–806. doi: 10.1210/jc.2017-00090 (PMC5546856; doi:10.1210/jc.2017-00090)

Supplementary material

**Methods**

*Assessment of hormone predictors*

IGF-1 and insulin-like growth factor binding protein 3 (IGFBP-3) were measured by chemiluminiscence immunoassay (Molecular Endocrinology, Faculty of Medicine, University of Santiago de Compostela, Spain). Within- and between-assay coefficients of variation (CV) were 7.4% and 2.9% for IGF-1, and 7.2% and 4.2% for IGFBP-3. Detection limits of the respective assays were 20 µg/L for IGF-1 and 0.1 μg/ml for IGFBP-3.

DHEA-S was measured using the Modular E170 platform electrochemiluminescence immunoassay (Roche Diagnostics, Mannheim, Germany). Intra- and inter-assay coefficients of variation (CVs) were 1.1% and 4.1%, respectively, and detection limit 0.003 µmol/L.

Serum 25OHD levels were determined by radioimmunoassay (DiaSorin, Stillwater, MN, USA), with intra- and inter-assay coefficients of variation (CV) of 11% and 8%, respectively, and detection limit of 2.0 ng/ml.

Serum PTH was assayed using a chemiluminescence immunoassay (Bio-Intact PTH, Quest Diagnostics, Madison, NJ, USA), with intra- and inter-assay CVs of 6% and 2.8%, respectively, and detection limit of 1.6 pg/ml.

| **Supplementary Table 1. The European Male Ageing Study frailty phenotype criteria and the original Cardiovascular Health Study criteria** | | | | | | |
| --- | --- | --- | --- | --- | --- | --- |
| **Criteria** |  | **Cardiovascular Health Study** | |  | **European Male Ageing Study** | |
|  |  | **Adopted Measure** | **Cut-point** |  | **Adopted Measure** | **Cut-point** |
| ***Exhaustion/***  ***Poor endurance*** |  | Centre for Epidemiological Studies Depression Scale;  Question: 'I felt that everything I did was an effort' or 'I could not get going' | Answered ' Most of the time' or ' A moderate amount of the time' |  | Beck Depression Inventory | Answered: ‘I don't have enough energy to do very much/anything' or  ‘I am too tired to do a lot of/ most of the things I used to do’ |
| ***Slowness*** |  | Walking time: 15 feet | Slowest 20% by gender and height |  | Walking time: 50 feet | Slowest height-adjusted quintile of participants aged>65 years:  ≥17.2 sec for height ≤173.5cm; ≥16 sec for height >173.5cm |
| ***Low activity*** |  | Minnesota Leisure Time Activity questionnaire;  Kilocalorie expenditure per week | Lowest 20% |  | Physical Activity Scale for the Elderly | Lowest quintile of participant aged > 65 years: score ≤78 |
| ***Weakness*** |  | Grip strength | Lowest 20% by gender and BMI |  | 5 Chair-stands test | Slowest 10% of participants aged >65: ≥17.8 sec or unable |
| ***Sarcopenia*** |  | Weight loss | > 10 pounds unintentional weight loss in the past 12 months |  | Mid-upper arm muscle circumference | Lowest 10% of participants aged >65 years: ≤ 23.7 cm |

| **Origin** | **Variable/ Item** | **Score** |
| --- | --- | --- |
| 36-Item Short Form Survey | Self-rated general health | Fair/ Poor =1, Good=0.5, Excellent/ Very good=0 |
|  | Feeding yourself | Limited / Limited a little=1, Not Limited=0 |
|  | Walking in your home | Limited / Limited a little=1, Not Limited=0 |
|  | Bathing and dressing yourself | Limited / Limited a little=1, Not Limited=0 |
|  | Walking 1 km | Limited =1, Limited a little=0.5, Not Limited=0 |
|  | Walking more than 1 km | Limited =1, Limited a little=0.5, Not Limited=0 |
|  | Climbing one flight of stairs | Limited =1, Limited a little=0.5, Not Limited=0 |
|  | Climbing several flights of stairs | Limited =1, Limited a little=0.5, Not Limited=0 |
|  | Ability to perform moderate activity | Limited =1, Limited a little=0.5, Not Limited=0 |
|  | Ability to perform vigorous activity | Limited =1, Limited a little=0.5, Not Limited=0 |
|  | In the past 4 weeks have you accomplished less than you would like as a result of your physical health | All/Most of time=1, Sometime=0.5, Little time/ None=0 |
|  | In the past 4 weeks have you cut down on the amount of time spent on work or other activities as a result of emotional problems | All/Most of time=1, Sometime=0.5, Little time/ None=0 |
|  | In the past 4 weeks, have you been feeling full of life | Little time/ None=1, Sometime=0.5, All/Most of time=0 |
|  | In the past 4 weeks, have you been feeling 'in the dumps' | All/Most of time=1, Sometime=0.5, Little time/ None=0 |
|  | In the past 4 weeks, have you been feeling downhearted | All/Most of time=1, Sometime=0.5, Little time/ None=0 |
|  | In the past 4 weeks, have you been feeling tired | All/Most of time=1, Sometime=0.5, Little time/ None=0 |
|  | During the past 6 months, have you experienced serious illness or injury to yourself | Yes=1, No=0 |
| Beck Depression Inventory | Change in sleep pattern | Sleeping less/ lot more=1, Sleeping more/same=0 |
|  | Concentration | Poor=1, Fair=0.5, As good as ever=0 |
| International Prostate Symptom Score | *Over the past month, how often have you had to:* |  |
|  | Postpone urination | Always, >50%=1, about or less than 50%=0.5, Not at all, <20%=0 |
|  | Night urinate | Twice or more =1, once or not at all =0 |
|  | Weak Stream | Always, >50%=1, about or less than 50%=0.5, Not at all, <20%=0 |
| Self-reported morbidities | Heart condition | Yes=1, No=0 |
|  | High blood pressure | Yes=1, No=0 |
|  | Bronchitis | Yes=1, No=0 |
|  | Asthma | Yes=1, No=0 |
|  | Diabetes | Yes=1, No=0 |
|  | Liver condition | Yes=1, No=0 |
|  | Kidney condition | Yes=1, No=0 |
|  | Prostate disorder | Yes=1, No=0 |
|  | Thyroid disorder | Yes=1, No=0 |
|  | Cancer (ever) | Yes=1, No=0 |
|  | Stroke (ever) | Yes=1, No=0 |
| Rey-Osterrieth Complex Figure (ROCF) | Copying | Score <28, =1, Score≥ 28, = 0 |
|  | Delayed reproduction | Score <8, =1, Score ≥ 8, = 0 |
| Camden Topographical Recognition Memory (CTRM) | Total score | Score <16, =1, Score≥ 16, = 0 |
| Digit-Symbol Substitution (DSST) test | Total score | Score <16, =1, Score≥ 16, = 0 |
| Physical Performance Test | Time to walk 15.4 meters | Time≥ 16.7, =1, Time < 16.7, = 0 |
| Tinetti test | Total score | Score < 25, =1, Score ≥25, = 0 |

**Supplementary Table 2. The list of health deficit variables comprising 39-item EMAS Frailty Index**

| **Supplementary Table 3. Baseline Characteristics of the Analytical Sample (men with complete data on FP), men who were lost to follow-up and men who died during follow-up.** | | | | | |
| --- | --- | --- | --- | --- | --- |
|  | **Status at follow-up** | | | | |
| **Baseline parameter** | **Participant** | **Lost to f-up** | **Died** | **p value** |  |
| N | 2114 | 435 | 188 |  |  |
| **Age, years** | 58±10 | 61±12^a^ | 69±8^ab^ | <.001 |  |
| BMI, kg/m2 | 27.6±4.0 | 27.8±4.3 | 27.7±5.0 | 0.530 |  |
| **WHR** | 0.98±0.06 | 0.99±0.06 | 1.0±0.06^a^ | 0.001 |  |
| Waist circumference, cm | 98.1±10.9 | 99±11.8 | 99.6±12.4 | 0.086 |  |
| **Smoking, n (%)** | 420(20) | 116(27) | 51(28) | 0.001 |  |
| Frequent Alcohol, n (%) | 501(24) | 93(22) | 33(18) | 0.212 |  |
| **Below degree education, n (%)** | 1506(71) | 218(52) | 94(53) | <.001 |  |
| **Systolic BP, mmHg** | 145±20 | 148±22^a^ | 151±25^ab^ | <.001 |  |
| Diastolic BP, mmHg | 87±12 | 88±13 | 85±15 | 0.167 |  |
| **Creatinine, μmol/L** | 92±29 | 94±54 | 100±38^ab^ | <.001 |  |
| **BDI total** | 6±6 | 8±7^a^ | 11±8^ab^ | <.001 |  |
| **Mild depression (BDI band 2-3), n (%)** | 347(17) | 88(22) | 51(32) | <.001 |  |
| **Severe depression (BDI band 4-6), n (%)** | 56(3) | 24(7) | 21(16) | <.001 |  |
| **Diabetes, n (%)** | 119(6) | 41(10) | 32(18) | <.001 |  |
| **DHEA-S, µmol/L** | 4.7±2.7 | 4.8±3.2 | 3.2±2.2^ab^ | <.001 |  |
| **PTH, pg/ml** | 28.4±14.5 | 30.4±15.6 | 34.2±26.7 | 0.029 |  |
| **25-hydroxy vitamin D, ng/ml** | 26.0±13.0 | 22.3±11.6^a^ | 19.0±11.1^ab^ | <.001 |  |
| **IGF-1, µg/L** | 135.6±43.1 | 130.0±44.0 | 117.1±48.0^ab^ | <.001 |  |
| **IGFBP-3, μg/mL** | 4.5±1.0 | 4.3±1.0^a^ | 3.8±1.2^ab^ | <.001 |  |
| **Frailty phenotype: Robust, n (%)** | 1589(75) | 252(66) | 62(39) | <.001 |  |
| **Prefrail, n (%)** | 505(24) | 116(30) | 79(50) |  |
| **Frail, n (%)** | 20(1) | 16(4) | 17(11) |  |
| **Sarcopenia, n (%)** | 99(5) | 31(7) | 25(14) | <.001 |  |
| **Exhaustion, n (%)** | 130(6) | 45(11) | 39(21) | <.001 |  |
| **Low activity, n (%)** | 189(9) | 54(14) | 40(23) | <.001 |  |
| **Weakness, n (%)** | 112(5) | 34(8) | 25(14) | <.001 |  |
| **Slowness, n (%)** | 135(6) | 51(12) | 70(38) | <.001 |  |
| Data are expressed as mean ± standard deviation for continuous variables or as number (percentage) for binary categorical variables. Abbreviations: BMI, body mass index; BP, Blood Pressure; BDI, Beck Depression Inventory score; DHEA-S, dehydroepiandrosterone sulphate; IGF-1, insulin-like growth factor 1; IGFBP-3, insulin-like growth factor binding protein 3; N, number; WHR, Waist to Hip Ratio  P values were calculated using analyses of variance or the Kruskall Wallis test for continuous variables and the chi-squared test for categorical variables.  a Data differ significantly (p<0.05) from those in the participant group on post-hoc analysis using Tukey-Kramer for continuous variables or the z-test for categorical variables with correction for multiple pairwise comparisons  b Data differ significantly (p<0.05) from those lost to follow-up on post-hoc analysis using Tukey-Kramer for continuous variables or the z-test for categorical variables with correction for multiple pairwise comparisons. | | | | |  |

| **Supplementary Table 4. Baseline Characteristics of the Analytical Sample (men with complete data on FI), men who were lost to follow-up and men who died during follow-up.** | | | | |
| --- | --- | --- | --- | --- |
|  | **Status at follow-up** | | | |
| **Baseline parameter** | **Participant** | **Lost to f-up** | **Died** | **p value** |
| N | 2444 | 435 | 188 |  |
| **Age, years** | 59±11 | 61±12^a^ | 69±8^ab^ | <.001 |
| BMI, kg/m2 | 27.6±4.0 | 27.8±4.0 | 27.7±5.0 | 0.510 |
| **WHR** | 0.98±0.06 | 0.99±0.06 | 1.0±0.06^ab^ | 0.002 |
| Waist circumference, cm | 98.3±10.8 | 99±11.8 | 99.6±12.4 | 0.421 |
| **Smoking, n (%)** | 474(20) | 116(27) | 51(28) | 0.001 |
| Frequent Alcohol, n (%) | 561(23) | 93(22) | 33(18) | 0.253 |
| **Below degree education, n (%)** | 1731(71) | 218(52) | 94(53) | <.001 |
| **Systolic BP, mmHg** | 146±20 | 148±22 | 151±25^ab^ | <.001 |
| Diastolic BP, mmHg | 87±12 | 88±13 | 85±15 | 0.305 |
| **Creatinine, μmol/L** | 92±28 | 94±54 | 100±38^ab^ | <.001 |
| **BDI total** | 6±6 | 8±7^a^ | 11±8^abc^ | <.001 |
| **Mild depression (BDI band 2-3), n (%)** | 398(17) | 88(22) | 51(32) | <.001 |
| **Severe depression (BDI band 4-6), n (%)** | 82(4) | 24(7) | 21(16) | <.001 |
| **Diabetes, n (%)** | 153(6) | 41(10) | 32(18) | <.001 |
| **DHEA-S, µmol/L** | 4.6±2.7 | 4.8±3.2 | 3.2±2.2^ab^ | <.001 |
| **PTH, pg/ml** | 28.3±14.2 | 30.4±15.6^a^ | 34.2±26.7^ab^ | 0.040 |
| **25-hydroxy vitamin D, ng/ml** | 25.9±12.9 | 22.3±11.6^a^ | 19.0±11.1^ab^ | <.001 |
| **IGF-1, µg/L** | 135.0±42.8 | 130.0±44.0 | 117.1±48^ab^ | <.001 |
| **IGFBP-3, μg/mL** | 4.5±1.0 | 4.3±1.0^a^ | 3.8±1.2^ab^ | <.001 |
| **Frailty Index** | 0.09±0.1 | 0.11±0.12^a^ | 0.23±0.14^ab^ | <.001 |
| Data are expressed as mean/median ± standard deviation for continuous variables or as number (percentage) for binary categorical variables  Abbreviations: BMI, body mass index; BP, Blood Pressure; BDI, Beck Depression Inventory score; DHEA-S, dehydroepiandrosterone sulphate; IGF-1, insulin-like growth factor 1; IGFBP-3, insulin-like growth factor binding protein 3; N, number; WHR, Waist to Hip Ratio  P values were calculated using analyses of variance or the Kruskall Wallis test for continuous variables and the chi-squared test for categorical variables.  a Data differ significantly (p<0.05) from those in the participant group on post-hoc analysis using Tukey-Kramer for continuous variables or the z-test for categorical variables with correction for multiple pairwise comparisons  b Data differ significantly (p<0.05) from those lost to follow-up on post-hoc analysis using Tukey-Kramer for continuous variables or the z-test for categorical variables with correction for multiple pairwise comparisons. | | | | |

| **Supplementary Table 5. Association between baseline level of anabolic hormone and 4-year % change in frailty index** | | | | | | | | | | | | | | | | | | | |
| --- | --- | --- | --- | --- | --- | --- | --- | --- | --- | --- | --- | --- | --- | --- | --- | --- | --- | --- | --- |
| Models and adjustments | | | | | | | | | | | | | | | | | | | |
| Baseline Parameter | N | Model 1  Baseline frailty | | | Model 2  Baseline frailty and age | | | Model 3  Baseline frailty, age and centre | | | Model 4  Baseline frailty, age, centre, BMI | | | Model 5  (Model 4+smoking, alcohol, education, PASE) | | | Model 6  (Model 5+all endocrine predictors+TT+E2) | | |
|  |  | **% change^a^** | **95% CI** | **P-value** | **% change^a^** | **95% CI** | **P-value** | **% change^a^** | **95% CI** | **P-value** | **% change^a^** | **95% CI** | **P-value** | **% change^a^** | **95% CI** | **P-value** | **% change^a^** | **95% CI** | **P-value** |
| **IGF-1** | 2426 | -7.2 | -9.3, -5.0 | <.001 | -4.1 | -6.2, -1.7 | 0.001 | -3.7 | -6.0, -1.5 | 0.001 | -3.7 | -6.0, -1.5 | 0.001 | -3.6 | -5.8 -1.2 | 0.003 | -2.5 | -4.9, 0.5 | 0.108 |
| **IGFBP-3** | 2428 | -8.2 | -10.5, -5.8 | <.001 | -4.8 | -6.9, -2.6 | <.001 | -3.5 | -5.7, -1.4 | 0.002 | -4.2 | -6.4, -2.0 | <.001 | -3.4 | -5.8, -1.0 | 0.005 | -1.7 | -4.7, -1.3 | 0.267 |
| DHEA-S | 2428 | -6.0 | -8.2, -3.6 | <.001 | 1.0 | -1.5, 3.7 | 0.437 | -0.1 | -2.7, 2.4 | 0.917 | 0.2 | -2.3, 2.8 | 0.852 | -1.0 | -3.6, 1.5 | 0.428 | -0.3 | -3.0, 2.0 | 0.808 |
| PTH* | 2429 | 2.2 | 0.1, 4.5 | 0.059 | 1.3 | -0.8, 3.6 | 0.230 | 1.3 | -0.8, 3.6 | 0.231 | 0.9 | -1.2, 3.1 | 0.402 | 0.9 | -1.2, 3.1 | 0.401 | 1.7 | -0.8, 4.3 | 0.182 |
| **25-hydroxy vitamin D**** | 2347 | -3.3 | -5.6, -1.0 | 0.006 | -4.7 | -6.9, -2.5 | <.001 | -4.7 | -7.0, -2.4 | <.001 | -4.4 | -6.7, -2.0 | <0.001 | -4.2 | -6.6, -1.8 | 0.001 | -4.0 | -6.3, -1.4 | 0.002 |
| * Model 3-5 additionally adjusted for baseline 25(OH) D level  **Model 3-5 additionally adjusted for baseline PTH level  Abbreviations: BMI, body mass index; DHEAs, dehydroepiandrosterone sulphate; E2, estradiol; IGFBP 3, insulin-like growth factor binding protein 3; IGF-1, insulin-like growth factor 1; N, sample size; PASE, Physical Activity Scale for the Elderly; PTH, parathormone; TT, Total Testosterone ^a^ Change (% change/ 4 years) in frailty index per standard deviation increase in anabolic hormone level. Negative % change means that the baseline hormone level is associated with improvement of frailty status and positive % change means that the hormone is associated with worsening of frailty status. | | | | | | | | | | | | | | | | | | | |

| **Supplementary Table 6. Multivariable-adjusted odds ratio (95% CI) for worsening frailty phenotype associated with baseline hormonal predictor** | | | | | | | | | | | | | | | | | | | |
| --- | --- | --- | --- | --- | --- | --- | --- | --- | --- | --- | --- | --- | --- | --- | --- | --- | --- | --- | --- |
| Models and adjustments | | | | | | | | | | | | | | | | | | | |
| Baseline Parameter | N | Model 1  Baseline frailty | | | Model 2  Baseline frailty and age | | | Model 3  Baseline frailty, age and centre | | | Model 4  Baseline frailty, age, centre, BMI | | | Model 5  (Model 4+smoking, alcohol, education, CVD, DM) | | | Model 6  (Model 5+all endocrine predictors+TT+E2) | | |
|  |  | **OR** | **95% CI** | **P-value** | **OR** | **95% CI** | **P-value** | **OR** | **95% CI** | **P-value** | **OR** | **95% CI** | **P-value** | **OR** | **95% CI** | **P-value** | **OR** | **95% CI** | **P-value** |
| **IGF-1** | 1885 | 0.77 | 0.68, 0.86 | <.001 | 0.85 | 0.75, 0.96 | 0.008 | 0.82 | 0.73, 0.93 | 0.002 | 0.82 | 0.73, 0.93 | 0.002 | 0.84 | 0.74, 0.95 | 0.006 | 0.85 | 0.72, 0.99 | 0.038 |
| **IGFBP-3** | 1888 | 0.81 | 0.73, 0.90 | <.001 | 0.90 | 0.80, 1.01 | 0.070 | 0.84 | 0.74, 0.94 | 0.003 | 0.84 | 0.75, 0.95 | 0.006 | 0.87 | 0.77, 0.98 | 0.025 | 0.98 | 0.83, 1.14 | 0.772 |
| DHEA-S | 1891 | 0.87 | 0.78, 0.97 | 0.012 | 1.07 | 0.95, 1.22 | 0.259 | 1.07 | 0.94, 1.21 | 0.299 | 1.06 | 0.93, 1.20 | 0.390 | 1.05 | 0.92, 1.19 | 0.473 | 1.07 | 0.94, 1.23 | 0.284 |
| PTH* | 1890 | 1.05 | 0.95, 1.16 | 0.329 | 1.03 | 0.93, 1.14 | 0.588 | 1.00 | 0.90, 1.12 | 0.938 | 1.01 | 0.90, 1.13 | 0.860 | 1.02 | 0.92, 1.13 | 0.726 | 1.06 | 0.92, 1.23 | 0.419 |
| **25-hydroxy vitamin D**** | 1828 | 0.92 | 0.83, 1.03 | 0.162 | 0.89 | 0.80, 1.00 | 0.053 | 0.86 | 0.76, 0.97 | 0.013 | 0.84 | 0.75, 0.95 | 0.007 | 0.85 | 0.75, 0.97 | 0.013 | 0.87 | 0.76, 0.98 | 0.027 |
| * Model 3-5 additionally adjusted for baseline 25(OH) D level  **Model 3-5 additionally adjusted for baseline PTH level  Abbreviations: BMI, body mass index; CVD, cardiovascular disease; DHEAs, dehydroepiandrosterone sulphate; DM, diabetes mellitus; E2, estradiol; IGFBP3, insulin-like growth factor binding protein 3; IGF-1, insulin-like growth factor 1; N, sample size; PTH, parathormone, TT, total testosterone | | | | | | | | | | | | | | | | | | | |

| **Supplementary Table 7. Multivariable-adjusted odds ratio (95% CI) for improving frailty phenotype associated with baseline hormonal predictor** | | | | | | | | | | | | | | | | | | | |
| --- | --- | --- | --- | --- | --- | --- | --- | --- | --- | --- | --- | --- | --- | --- | --- | --- | --- | --- | --- |
| Models and adjustments | | | | | | | | | | | | | | | | | | | |
| Baseline Parameter | N | Model 1  Baseline frailty | | | Model 2  Baseline frailty and age | | | Model 3  Baseline frailty, age and centre | | | Model 4  Baseline frailty, age, centre, BMI | | | Model 5  (Model 4+smoking, alcohol, education, CVD, DM) | | | Model 6  (Model 5+all endocrine predictors+TT+E2) | | |
|  |  | **OR** | **95% CI** | **P-value** | **OR** | **95% CI** | **P-value** | **OR** | **95% CI** | **P-value** | **OR** | **95% CI** | **P-value** | **OR** | **95% CI** | **P-value** | **OR** | **95% CI** | **P-value** |
| IGF-1 | 471 | 1.08 | 0.91, 1.29 | 0.377 | 0.96 | 0.79, 1.15 | 0.643 | 0.94 | 0.78, 1.15 | 0.555 | 0.94 | 0.77, 1.14 | 0.539 | 0.93 | 0.76, 1.14 | 0.499 | 0.89 | 0.67, 1.17 | 0.405 |
| IGFBP-3 | 471 | 1.21 | 1.00, 1.46 | 0.049 | 1.06 | 0.87, 1.29 | 0.579 | 1.04 | 0.85, 1.28 | 0.686 | 1.02 | 0.83, 1.25 | 0.863 | 1.04 | 0.84, 1.29 | 0.720 | 1.08 | 0.79, 1.47 | 0.621 |
| DHEA-S | 471 | 1.19 | 0.98, 1.45 | 0.072 | 0.91 | 0.73, 1.13 | 0.396 | 0.94 | 0.75, 1.17 | 0.589 | 0.95 | 0.76, 1.18 | 0.640 | 0.98 | 0.78, 1.25 | 0.906 | 1.04 | 0.80, 1.35 | 0.758 |
| PTH* | 472 | 1.03 | 0.84, 1.26 | 0.775 | 1.08 | 0.87, 1.33 | 0.476 | 1.09 | 0.87, 1.37 | 0.452 | 1.08 | 0.86, 1.36 | 0.481 | 1.07 | 0.84, 1.35 | 0.591 | 1.09 | 0.85, 1.39 | 0.482 |
| 25-hydroxy vitamin D** | 459 | 1.14 | 0.94, 1.37 | 0.172 | 1.18 | 0.97, 1.44 | 0.099 | 1.25 | 0.99, 1.56 | 0.051 | 1.27 | 1.01, 1.58 | 0.039 | 1.23 | 0.98, 1.56 | 0.077 | 1.24 | 0.98, 1.57 | 0.073 |
| * Model 3-5 additionally adjusted for baseline 25(OH) D level  **Model 3-5 additionally adjusted for baseline PTH level  Abbreviations: BMI, body mass index; CVD, cardiovascular disease; DHEAs, dehydroepiandrosterone sulphate; DM, diabetes mellitus; E2, estradiol; IGFBP3, insulin-like growth factor binding protein 3; IGF-1, insulin-like growth factor 1; N, sample size; PTH, parathormone, TT, total testosterone | | | | | | | | | | | | | | | | | | | |


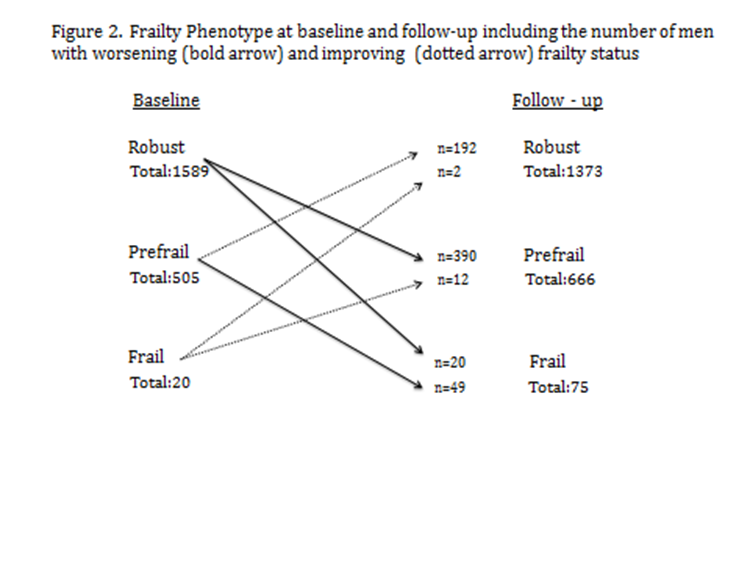


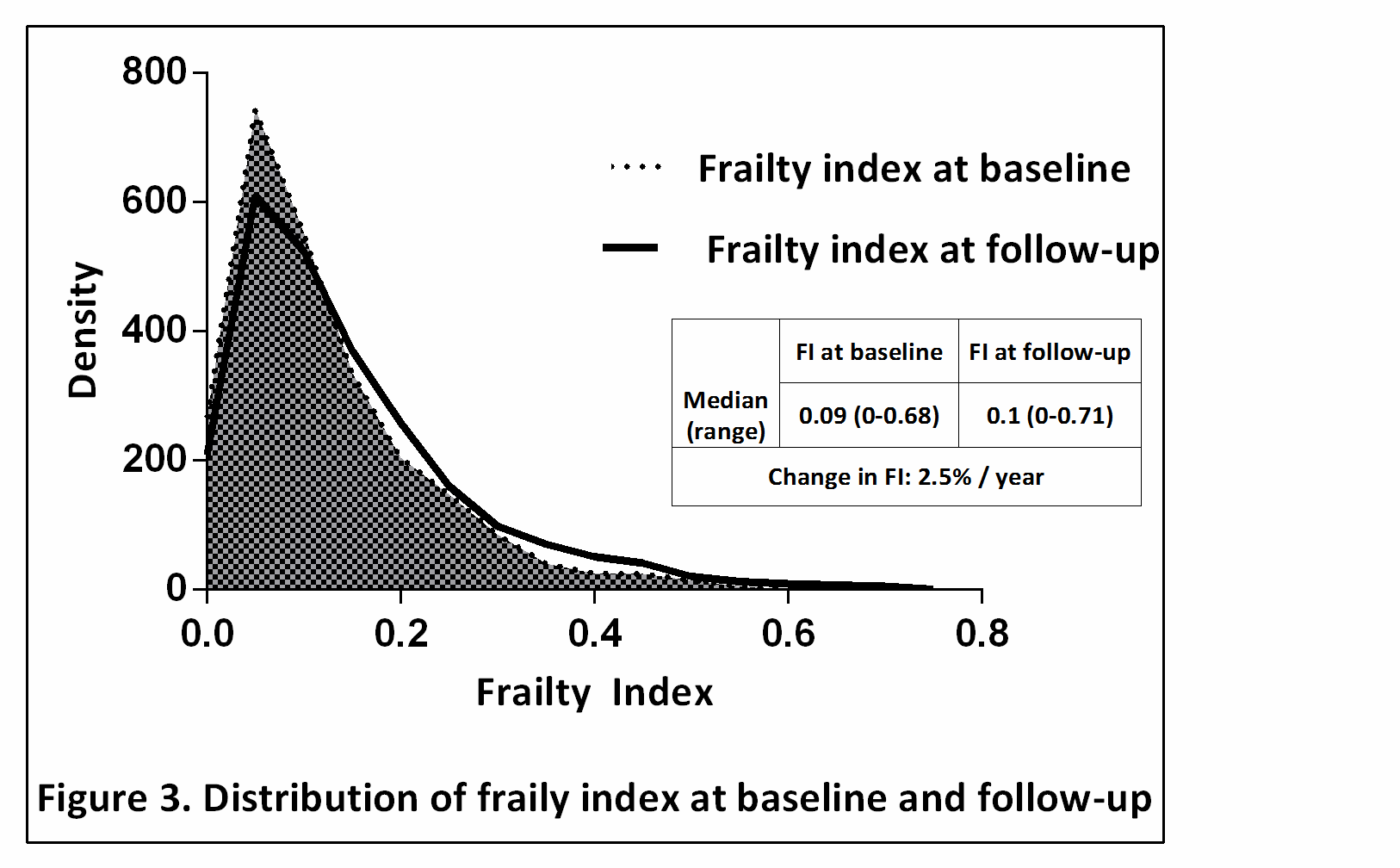


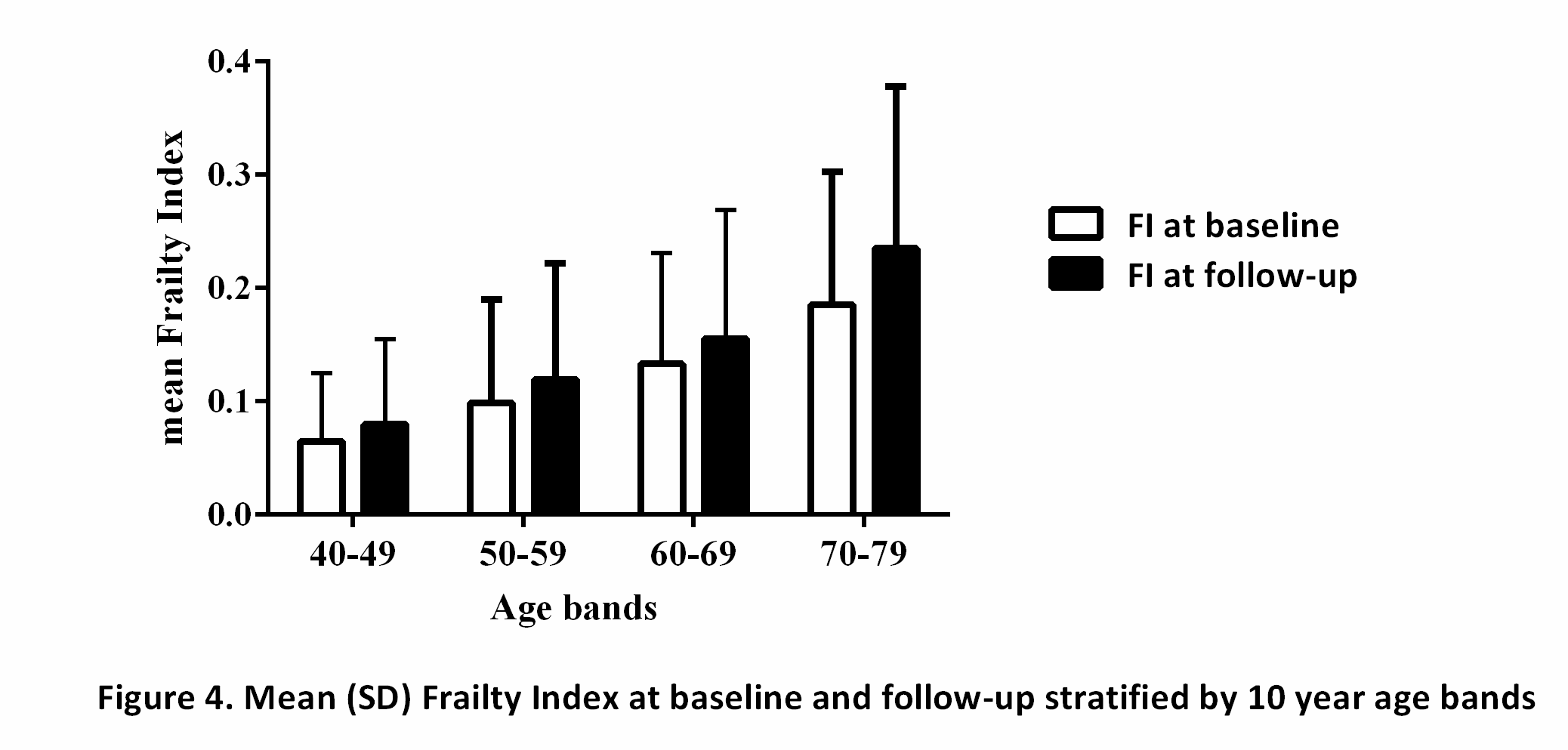


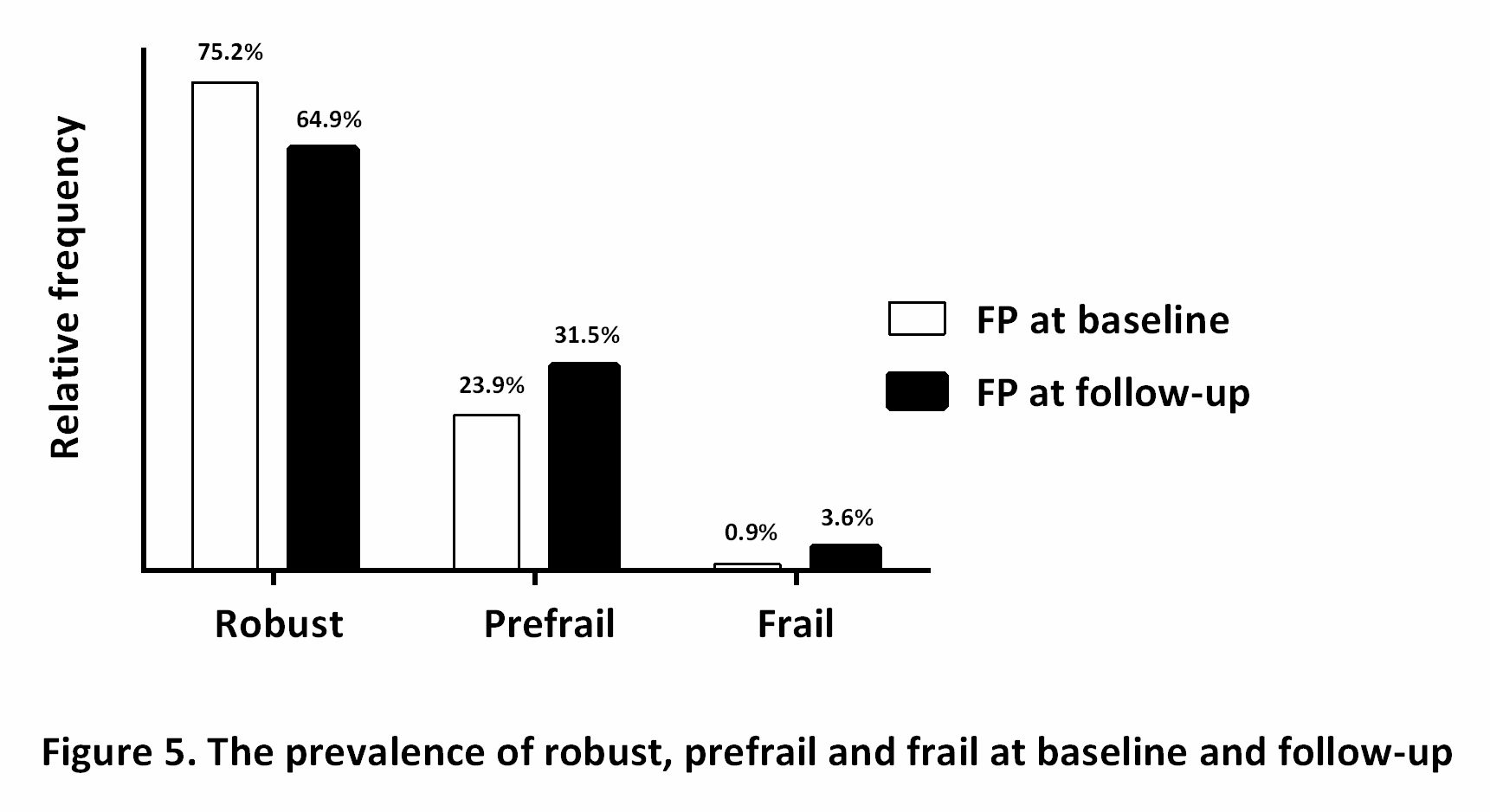

Supplement: Supplementary file 1 [file jc.2017-00090.sm1.doc]
